# Stakeholder Involvement in the Governance of Human Genome Editing in Japan

**DOI:** 10.1007/s41649-023-00251-8

**Published:** 2023-04-26

**Authors:** Tatsuki Aikyo, Atsushi Kogetsu, Kazuto Kato

**Affiliations:** 1 Department of Biomedical Ethics and Public Policy, Graduate School of Medicine, Osaka University, Osaka, Japan; 2 Graduate School of Biomedical and Health Sciences, Hiroshima University, Hiroshima, Japan

**Keywords:** Human genome editing, Human embryo research, Expert panel on Bioethics, Japan, Ethics review, Governance, Stakeholder involvement

## Abstract

Genome editing is a technology that can accurately and efficiently modify the genome of organisms, including the human genome. Although human genome editing (HGE) has many benefits, it also involves technical risks and ethical, legal, and social issues. Thus, the pros and cons of using this technology have been actively debated since 2015. Notably, the research community has taken an interest in the issue and has discussed it internationally. However, for the governance of HGE, the roles of government agencies and the general public are also important for an effective regulatory system. Here, we examine the roles of the research community, government, and public in the governance of HGE through an analysis of discussions in the Japanese Expert Panel on Bioethics. During the discussion of the research ethics review system, the professionalism of the research community and the pros and cons of state oversight have become issues for debate. Furthermore, through an examination of the overall policy-making process, three stakeholders are clearly involved in the governance of emerging medical technologies in the Expert Panel on Bioethics, a discussion forum established by government agencies. The contrast among these roles provides insight into the positive roles of government agencies and the research community and the conditions under which these roles are played. We also note that there are diverse actors in the public, which may have an impact on their participation. Our results may serve as a guide for countries and organizations to establish governance on emerging medical technologies.

## Introduction

In several countries, the treatment of human embryos for medical research has long been debated from ethical and social perspectives. Whether research involving human embryos is acceptable has been regulated differently in each country, reflecting differences in social backgrounds and attitudes. On the one hand, the United Kingdom has a long history of research in embryology and stem cell biology; for example, the world’s first in vitro fertilization baby was born in the United Kingdom in 1978 (Steptoe and Edwards 1978). This has led to the establishment of the Human Fertilisation and Embryo Authority (HFEA), a regulatory authority that controls research and clinical practice of human embryos, in 1990 (Lovell-Badge 2008). On the other hand, in the USA, federal funding was greatly restricted by the so-called Dickey–Wicker amendment that was introduced in 1996, and there was little federal regulation; thus, guidelines were developed by scientific organizations, and these are operating well (Hynes 2008).

In recent years, the development of genome editing technologies has led to an increased interest in human embryo research. Genome editing technology using the CRISPR/Cas9 system was developed in 2012 (Jinek et al. 2012), for which Emmanuelle Charpentier and Jennifer A. Doudna were awarded the Nobel Prize in Chemistry in 2020. This breakthrough technology excels in accuracy and efficiency, and its potential for application in medical research, that is, the broad applicability of genome editing of human DNA, has become a reality (National Academy of Sciences, Engineering, and Medicine 2017). This has led to the emergence of various ethical, legal, and social issues (ELSI). The issues associated with human embryo genome editing technology are also relevant to conventional human embryo research. As a result, in the discussions on human genome editing, ethical issues on the use of human embryo, and thus germ cells, are separately considered from those on somatic genome editing. However, some people have pointed out that since safety is the only thing currently supporting this distinction, this will immediately cease to exist once germline HGEs become safe (Evans 2021).

With the advent of genome editing technologies, the need for establishing proper research governance has arisen. Tools for the effective governance of human genome editing, such as declarations, treaties, conventions, legislation, and regulations, and organizations responsible for governance, such as national science and medicine societies and institutions, professional self-regulation, public advocacy, and activism, (World Health Organization 2021) have been pointed out. In other words, multiple actors play different roles not only in government agencies, but also in non-government agencies. For example, the research community has discussed with interest the use of genome editing technologies for human embryos (Kaiser and Normile 2015). Scientific self-regulation helps clarify the responsibilities of scientists, implications of their research, and potential reactions from the public (Gregorowius et al. 2017). However, experts have been criticized for dismissing public views as simply ill-informed (Jasanoff et al. 2015). Others have argued that the role of government entities is also important for an effective regulatory system (Lei and Qiu 2020). Furthermore, the role of the general public in genome editing governance has also received attention (National Academy of Sciences, Engineering, and Medicine 2017). Thus, in light of the advent of genome editing technologies, the roles of stakeholders in research governance, including the research community, government, and public, are being questioned. However, presently, there is no consensus on the desired involvement of each actor, and further research on the involvement of these actors in research governance is required.

Studies on human embryos using genome editing in the countries that allow this kind of research are governed by a research ethics review system (hereafter, the ethics review system). The HFEA in the UK and the Embryonic Stem Cell Research Oversight Committee (ESCRO) in the USA are well-known ethics review systems for human embryo research. On the one hand, HFEA is characterized as a national review system that handles everything from basic research to clinical applications involving human embryos. On the other hand, ESCRO is an ethics review system established at the local level for basic research.

The ethics review systems for basic research in the medical and life sciences in Japan differ from those of the UK and the USA. In Japan, two main methods are used for ethics review: one requires only a review of the research plan by the Institutional Review Board (IRB), whereas the other is a “two-step review,” in which the research plan is reviewed by the IRB and confirmed by the national government. Generally, the former review system has been adopted; however, government guidelines for some human embryo studies require a two-step review. Although the two-step review system can compensate for the inadequacies of IRB reviews, it can further complicate the procedure. In addition to the review system for basic research regulated by the guidelines, there are review systems for clinical research and regenerative medicine regulated by law. In these review systems, a governmental certification system for IRBs is adopted to ensure their performance.

Thus, actual ethics review systems are diverse. There are differences according to the circumstances of each country and characteristics of the research. In Japan, consideration has been given to creating an ethics review system that is appropriate for the characteristics of human embryo genome editing technology. As previously mentioned, the roles of various stakeholders in the governance of genome editing are attracting attention. Considering this, the policy-making process of this new ethics review system for human embryo genome editing research deserves attention. However, to our knowledge, studies focusing on the ethics review system for human embryo genome editing research and its policy-making process, especially those considering the involvement of various stakeholders, are limited.

**Fig. 1:**
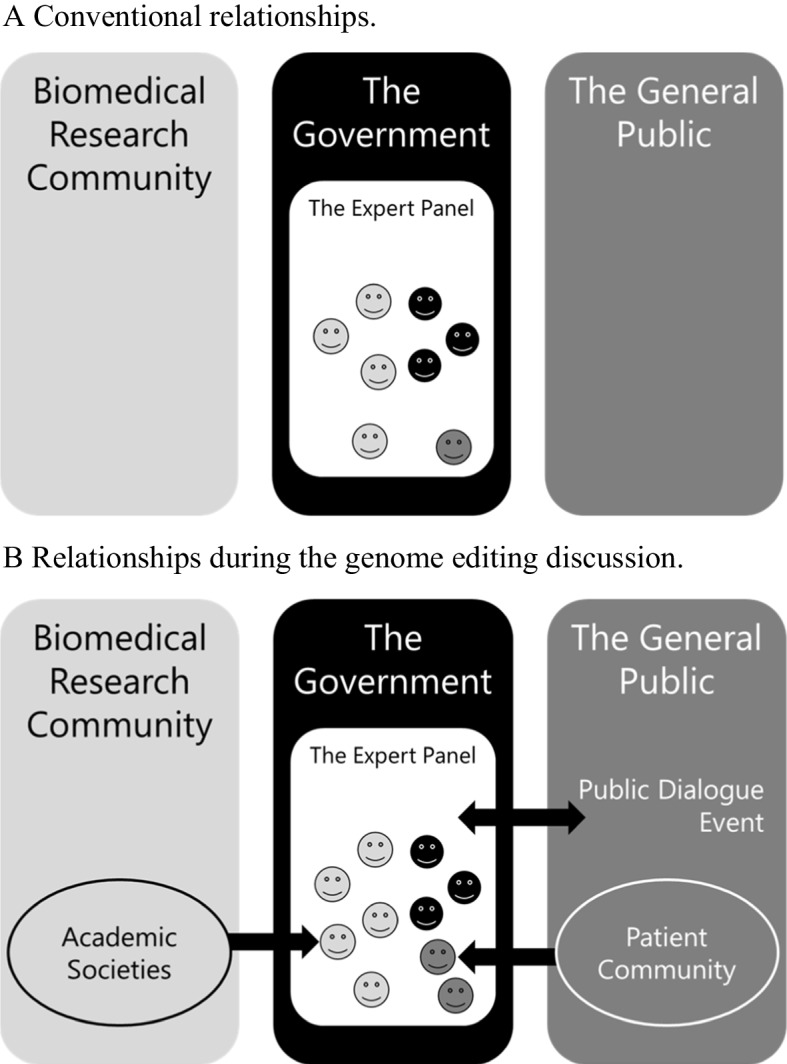
Three stakeholders and their discussion forum

In these discussions, national and supranational councils deserve attention because they play an important role in policy-making that influences governance. In Japan, the Expert Panel on Bioethics, the nation’s highest policy-making body on bioethics, was established at the Council for Science, Technology, and Innovation in 2001. Its main mission is to respond to developments in life sciences, and since its establishment, it has primarily been involved in discussions concerning the human embryo. In effect, it is a forum for discussing emerging medical technologies, including genome editing research. The Expert Panel, a governmental body, is a forum for discussion that informs national policy and is moderated by the government secretariat, and discussions are conducted by the expert members. According to the operating rules, the Expert Panel must comprise experts in the relevant fields and members to express their opinions from a general standpoint. Members are not required to represent their respective communities in the discussions; they participate in the discussions as individuals with their own attributes (Fig. 1-A). Since its inception, the Expert Panel has included experts from a wide variety of disciplines. The diversity of members may provide opportunities for diverse stakeholders to play different roles. In addition, we were able to review the discussions, as detailed minutes of the proceedings are available to the public. The Expert Panel on Bioethics has discussed many things from 2016–2020, including the governance of research and whether to conduct research on each research subject. Regarding the governance of research, while there are many elements involved in the governance of research, the Expert Panel’s discussion focused particularly on the ethics review system. This discussion also examined the stakeholders involved in the ethics review system and their roles.

In this review, we aim to identify the roles of the biomedical research community, government, and public in the governance of human genome editing through an analysis of the discussions by the Japanese Expert Panel on Bioethics and to gain insights into the nature of each role. The biomedical research community is comprised of scientists and engineers specialized in biomedical research. By government, we mean responsible national organizations such as the Cabinet Office, Ministry of Health, Labour and Welfare (MHLW), and Ministry of Education, Culture, Sports, Science and Technology (MEXT). By public, we mean citizens and other people who are not specialists in biomedical research nor academic researchers in humanities and social sciences. For the purpose of this study, we focused on two separate but related aspects of the activities of the Expert Panel on Bioethics. First, this study clarifies the process of discussions on the ethics review system for human embryo genome editing; we analyzed the roles of the research community, government, and public in the ethics review system. This level of analysis is useful for providing a specific and detailed analysis of the roles of the stakeholders. Second, we analyzed how each stakeholder was involved in the overall policy-making process during the period in which the Expert Panel on Bioethics dealt with genome editing research. The Expert Panel on Bioethics discussed not only the ethics review system, but also the extent to which genome editing research should be allowed. While the ethics review system is a relatively administrative issue, genome editing itself involves a variety of stakeholders, including those not directly involved in research or oversight. This level of analysis is crucial to obtain a full picture of the roles of all relevant stakeholders in the governance of genome-editing research. In particular, it is imperative to analyze how stakeholders in the nongovernmental sectors play their roles not only in the ethics review system, but also in the overall discussions and debates on the governance of the technology. This study is expected to have implications for the governance of emerging medical technologies.

## Methods

The subject of analysis in this study was the discussion of the Expert Panel on Bioethics.^1^ The minutes of the Expert Panel on Bioethics meetings are publicly available and suitable for the subject of the study as a source of information that can be obtained objectively. Presentation materials and related reports from the meetings were also referred to understand the discussions.

Discussions on human genome editing in the Expert Panel on Bioethics began at the 89th meeting (June 3, 2015), whereas discussions on the ethics review system for basic research using genome editing technology on human embryos began on April 22, 2016, with the “Research Using Genome Editing Technology on Human Fertilized Embryos (Interim Summary)” and was compiled as “Summary of Opinions on the Examination System” on January 10, 2020. During this period, the Task Force on the Review of the “Basic Principles on Handling of Human Embryos” (hereafter, ‘Task Force’) was established under the Expert Panel to actively discuss issues related to the rise of genome editing technologies. Therefore, the analysis covers the period from April 22, 2016, to January 10, 2020, and includes a total of 36 meetings held by the Expert Panel on Bioethics and the Task Force during the inclusion period.

One of the authors of this study (KK) participated in the discussions and reported writing as an expert member of the Expert Panel on Bioethics from 2010 to 2020. However, in this paper, he was involved in the objective analysis of the overall discussions of the Panel.

The subject matter of the discussions of the Expert Panel on Bioethics is wide-ranging. In accordance with the purpose of this study, we focused on the history of the discussions during the period under review, particularly the discussions on the ethics review system for human embryo genome editing research. Next, we analyzed the involvement of various stakeholders, including the research community, government, and the public, in both the ethics review system and overall policy-making process, based on the minutes of the proceedings.

## Results

### Summary of the Discussion of the Expert Panel on Bioethics

#### Organization of the Expert Panel

The Expert Panel and Task Force members were selected and convened by the Secretariat of the Expert Panel. These two committees always have at least 10 members and are organized according to their operating rules. According to the operating rules, the Expert Panel must have experts in the relevant fields and, indeed, medical, legal, and ethics experts participated in the committees. All of them are required to be experts, but they are not in a position to represent the organizations. They included members of the biomedical research community. There is also a need for members to express their opinions from the general standpoint and, therefore, journalists participated in the Expert Panel and representatives of patient organizations participated in the Task Force. The “general standpoint” here is intended to incorporate the viewpoints of non-professionals into the discussion, but is not representative of the general public.

There was no extreme bias in the male-to-female ratio of the participants throughout the study period. At the 98th Expert Panel (first meeting of the study period), of the fifteen members, seven were men, and eight were women; at the first Task Force, of the fourteen members, ten were men, and four were women.

#### Course of Discussion

The analysis covers four years of discussion in the Expert Panel on Bioethics. Based on the composition of the meeting body and timing of the report formulation, the entire period under analysis can be divided into four periods. Here, we mainly present the course of the discussions on the ethics review system and refer to some other discussion topics (Table 1).

**Table 1:** Four phases of meetings of the Expert Panel on Bioethics

	Period	Main meeting body^a^	Reports	Main discussions on the review system
First period	Expert Panel 98th (June 1, 2016) – 103rd (April 10, 2017)	Expert Panel	None	Involvement of academic societies
Second period	Expert Panel 104th (May 19, 2017) – 107th (March 9, 2018) Task Force 1st (July 14, 2017) – 6th (December 20, 2017)	Task force	First Report^b^	Possibilities other than “two-step review”
Third period	Expert Panel 108th (May 14, 2018) – 116th (June 12, 2019) Task Force 7th (May 14, 2018) – 18th (April 15, 2019)	Expert Panel and Task Force	Second Report^c^	Use of third-party organizations, central IRB, and HFEA
Fourth period	Expert Panel 117th (July 17, 2019) – 121st (December 18, 2019)	Expert Panel	Summary of Opinions^d^	Review of existing ethics review system and involvement of the government

^a^Main meeting body where discussions on genome editing were held

^b^First Report on the revision of the “Basic Principles on the Handling of Human Embryos”

^c^Second Report on the revision of the “Basic Principles on the Handling of Human Embryos”

^d^Summary of Opinions on the Review System of Basic Research Using Genome Editing Technology on Human Fertilized Embryos

In April 2016, the Expert Panel on Bioethics formulated a report titled “Research on the Use of Genome Editing Technology on Human Fertilized Embryos (Interim Summary).” At this time, no national guidelines for basic research on the use of genome editing technology in human embryos existed; therefore, this report became the starting point for discussion. At the 98th Expert Panel on Bioethics held immediately after the formulation of the Interim Summary, it was pointed out that the ethics review system for genome editing research should be discussed. In the ensuing discussion, methods such as using a two-step review process or involving academic societies were suggested, since the quality of the review process is not always sufficient for institutional ethics review boards. The period during which these discussions were held by the Expert Panel on Bioethics is referred to as the first period.

At the 104th meeting of the Expert Panel on Bioethics, the establishment of a Task Force was decided. The period up to the formulation of the First Report (107th meeting) is referred to as the second period. The Task Force discussed the policies for an ethical review system. It was decided that a consistent system that is not divided according to research purpose should be established, and the system should be regulated by guidelines rather than by law. It was also confirmed that a “two-step review” has been conventionally used for human embryo research; however, a review system other than this should be considered. Based on this discussion, the First Report concluded that it is appropriate to adopt a “two-step procedure” for the review system. This term is distinct from a “two-step review” in that it leaves open the possibility of adopting methods other than ethics review, such as notification to an administrative agency, as the second-step procedure. In addition, during this period, The Expert Panel discussed whether it is permissible to use human embryos for basic medical research using genome editing technology. The discussion was carried out step by step for different purposes of research, and whether surplus or newly created embryos were used. The first topic of discussion was research on assisted reproduction using surplus embryos, and the First Report stated that such research should be permitted provided that the individual research protocol is properly reviewed. A separate paper is currently being prepared for policy development in human embryo research.

In the third period, a wide range of proposals, not limited to the “two-step procedure,” were put forward. The use of third-party organizations and establishment of a central IRB were discussed, and an example of HFEA in the UK was repeatedly referred to. The Second Report stated that it would be appropriate for MEXT and MHLW to promptly develop guidelines and establish a rigorous review system that can make appropriate judgments regarding the acceptability of individual research plans and can pay attention to the actual situation in Japan and overseas, especially the current status of operation of the genome editing guidelines and other guidelines for research using human fertilized embryos. Moreover, disease research using surplus embryos and assisted reproductive medical research using embryos created for research purposes were discussed. The Second Report stated that such research should be permitted, provided that the individual research protocol is properly reviewed.

Subsequent discussions were held by the Expert Panel on Bioethics. The subject was the review system, and the report “Summary of Opinions on the Review System for Basic Research Using Genome Editing Technology on Human Fertilized Embryos” was compiled. This is referred to as the fourth period. In this period, an appropriate review system for basic research using genome editing technology on fertilized human embryos was discussed with reference to the existing ethics review system. It is also noteworthy that the government’s involvement in the review system has been discussed.

Thus, the ethics review system for research on the use of genome editing technologies in human embryos has been discussed intermittently over the four years.

### Ethics Review System

#### New Proposals for the Ethics Review System

The discussion on human embryo genome editing not only explored the possibility of applying the existing research ethics review system, but also extended it to the possibility of establishing a new review system based on unprecedented ideas, such as cooperation with academic societies and a centralized review. Existing ethics review systems in Japan include a method in which only an institutional ethics review board reviews the research plans and a “two-step review” method in which the national government, in addition to the institutional review board, conducts a review. However, in the former method, it is difficult to ensure the quality of the IRB's review, while in the latter method, the complexity of the procedure has been an issue. The Expert Panel has sought a way to resolve these issues.

During the first period, a proposed review structure involving academic societies was discussed with the Japan Society for Gene and Cell Therapy and other academic societies. The Japan Society for Gene and Cell Therapy has a strong interest in human genome editing, and in 2015, it issued a joint statement with the American Society for Gene and Cell Therapy (Friedmann et al. 2015). In addition, the society called on three other Japanese societies that may be involved in human embryo genome editing research. On April 22, 2016, it issued a “Statement from Four Societies on Human Genome Editing,” which assumes the need for discussion on ELSI for human genome editing and clarified its stance to actively engage in information and awareness-raising activities in collaboration with each other.^2^ The statement also argued that guidelines should be carefully and promptly prepared for the implementation of basic research on genome editing using human germ cells and embryos. In Japan, guidelines for basic research have served the function of prescribing details of the ethics review system. Representatives of these four relevant academic societies, which include many members who utilize genome editing technology, attended the 98th Expert Panel on Bioethics (June 1, 2016) to provide an explanation of the statement and comment on the interim summary from the standpoint of the societies. At this time, the Expert Panel and representatives of the four societies confirmed that there are areas in which the government should be involved and areas in which academic societies and others should be involved in the regulatory framework for human embryo genome editing. Representatives of the four societies reiterated the point made in the statement that the national government should first establish guidelines.

Conventional regulation of medical research in Japan has been carried out in various ways, including through laws and regulations, administrative guidelines, and self-regulation by academic societies. In discussions of the Expert Panel, it has been pointed out that while self-regulation by academic societies is more flexible than laws and administrative guidelines, there are some issues, such as the fact that they are not effective for those who are not members of an academic society. The four societies argued that both regulations prepared by the government and self-regulation by academic societies should be used to strengthen the overall governance of technology.^3^ The representatives of the societies stated that the government should prepare broad guidelines and the societies should be involved in scientific matters, so that both parties can play complementary roles.^4^ The Expert Panel on Bioethics agreed to this policy, and thereafter, a collaboration between the government and academic societies was sought.

The Expert Panel repeatedly pointed out the current lack of assurance of the quality of IRB reviews and the difficulty of achieving a certain level of review in all IRBs. Therefore, the first method of collaboration was to create a manual or guidelines to support IRB reviews. As the second method of collaboration, a proposal was made for a system in which academic societies are directly involved in the review process to ensure the quality of the review. This new review system had not been considered previously. In addition, the idea of the two-step review process involved establishing a central ethics review board (CRB). On the other hand, the actual discussion also reaffirmed the importance of the IRB, which is in a position close to the researchers and research facilities.

At the 101st Expert Panel on Bioethics meeting (October 21, 2016), three proposals were discussed regarding the division of roles between the government and academic societies.^5^ All three proposed establishing a CRB involving academic societies. One of them also proposed utilizing the conventional IRB in addition to the CRB. The other two proposed that the IRB observe the progress of the research plans, which they called “monitoring,” rather than conduct ethics reviews. The three proposals were distinguished according to the roles played by the CRB and IRB (Table 2). To implement these proposals, the four societies were preparing to establish a “Joint Committee on Genome Editing Research.”

**Table 2:** Three proposals for the ethics review system

	Role of CRB involving academic societies	Role of IRB
Proposal A	Review of research plans	“Monitoring”
Proposal B	Review of research plans, “Monitoring”	“Monitoring”
Proposal C	Second review	Primary review of research plans

However, at the 104th meeting, the Expert Panel on Bioethics and the four societies diverged on the policy of the review system, and these proposals were effectively withdrawn on paper. At the 104th Expert Panel on Bioethics, the importance of continuing to consider cooperation with academic societies was pointed out, and the participation of representatives of academic societies was also obtained. The government and the academic societies continued to engage in discussions on the ethics review system.

Furthermore, during the third period, discussions were held with reference to the HFE Act and the HFEA in the United Kingdom. Compared to the Japanese review system, the UK’s review system is unique in that it is centralized in HFEA and comprehensively covers both basic research and clinical applications. In particular, the centralized review system has been an ongoing topic of discussion in the Expert Panel on Bioethics. The CRB included in the proposal for the 101st meeting was expected to play a centralized role, which is the same role that the HFEA plays. In addition to centrality and comprehensiveness, the HFEA provides substantial guidance on the review process.

These discussions were not immediately reflected in the ethics review system. However, the Second Report summarized and published these discussions as follows.^6^

“The Expert Panel on Bioethics will consider the review procedures for research that involves genome editing, etc., with a view to utilizing a third-party organization (assumed to be an organization separate from each research institution or the national government) and collaborating with related academic societies, etc. In doing so, it is appropriate to pay attention to the actual situation in Japan and abroad, especially the current status of operation of the Guidelines for Genome Editing and other guidelines for research using human fertilized embryos, etc.”

In the Summary of Opinions, it was mentioned that “the government should prepare guidance (guideline commentary) that better responds to the needs of the fields of review, with reference to the approach of the HFEA in the UK, etc.”^7^

#### Involvement of the Government

In the fourth period, the pros and cons of government involvement in the ethics review process were discussed. Government involvement in the ethics review process is a conventional practice in Japan. However, there has been insufficient consideration of the advantages and disadvantages of such involvement.

At the 119th Expert Panel on Bioethics (October 9, 2019), a legal scholar and physician pointed out the following:^8^

- Although a legal basis is required for guideline conformity review, the language of the Genome Editing Guideline does not provide a basis for granting a comprehensive ethics review authority to a government agency.
- As a review by a government agency focusing on the content of research poses a major constitutional problem, in relation to academic freedom, it is necessary to establish very specific and clear criteria for the subject of review, if such content regulation is to be implemented.

According to MEXT’s explanation, the articles of the Genome Editing Guidelines describe the subject matter of the review as specific items, such as the purpose of the research, method of obtaining embryos, and informed consent. However, this explanation did not address all the issues raised. Another panel member who specializes in jurisprudence, while largely affirming the points made by this jurist and physician, touched on the difficulty of writing ethics into the article. He also pointed out the polysemous nature of the term ethics and distinguished between “ethics that enter into the inner mind” and “guidelines for professional conduct.” This led to a discussion of ethics in which the government may be involved, and while there are challenges when they pertain to “ethics that enter into the inner mind,” the “guidelines for conduct” are not considered inappropriate worldwide. After much discussion, the members of the Expert Panel set a goal to determine what is feasible and establish guidelines for conduct that most parties can agree upon. The legal deficiencies pointed out were overcome by this interpretation and did not result in changes in the text of the statute.

Throughout the four years of discussions, matters that had been taken for granted in the existing ethics review system were questioned from various aspects. As a result, a new ethics review system was not immediately established. However, new ideas were included in the report, and the issues raised provided fresh interpretations of the existing system.

### Involvement of Various Stakeholders in the Overall Discussions of the Expert Panel

The previous sections analyzed the discussions regarding the roles of various stakeholders in the review system. In this section, we analyze the roles of various stakeholders in the overall process of discussions by the Expert Panel on Bioethics. We focus on the research community and the general public, including patients, as new ways of engaging with them have been observed.

#### Research Community

The Expert Panel on Bioethics is an expert panel established by the Council for Science, Technology, and Innovation and the Cabinet Office, Government of Japan. In this Panel, discussions were held with the participation of a wide range of positions, from experts in diverse fields, including medicine and law, to those who represent the views of the general public, such as journalists. However, these experts were not necessarily able to represent the views of the professional organizations, such as academic societies.

More active involvement of the research community is expected in the discussion regarding research on the use of genome editing technologies in human embryos. The Interim Summary formulated on April 22, 2016, states, “We expect the research community to actively lead the discussion in an open manner from a broad scientific, ethical, and social perspective.”

On the same day, the four relevant academic societies published their statements on human genome editing. This led to the involvement of representatives of the four societies in discussions that followed the 98th Expert Panel on Bioethics. Although the proposed review system discussed at that time was scrapped relatively early, the relationship between the Expert Panel on Bioethics and academic societies was sustained thereafter. In the second period, the President of the Japan Society for Gene and Cell Therapy and the President of the Japan Society of Human Genetics were appointed as extraordinary members of the Task Force.

In the second period, an analogous process can be noted in the relationship between the Science Council of Japan, a Japanese academy, and the Expert Panel on Bioethics. On September 27, 2017, the Science Council of Japan released a statement entitled “The State of Genome Editing Technology in the Medical and Pharmaceutical Fields in Japan.” At the 4th Task Force held immediately after, the chairperson of the committee who took charge of preparing this statement was invited to the meeting. This person became a member of the Expert Panel on Bioethics and the Task Force in the third period.

The involvement of the medical community was confirmed during the third period, when human embryo genome editing for disease research was the subject of discussion. In February 2018, the Expert Panel on Bioethics inquired into the Japanese Association of Medical Sciences on research using genome editing technology in human fertilized embryos. The Vice President of the Japanese Association of Medical Sciences presented a progress report at the 12th Task Force (October 22, 2018) and a final report at the 18th Task Force (April 15, 2019). Based on the current state of research, the report focused on the scientific rationale for the use of genome editing technology in fertilized human embryos to potentially contribute to the etiology and developmental mechanisms of diseases, focusing on specific diseases or groups of diseases. According to the Japanese Association of Medical Sciences, the research community involved in disease research, there are disease groups for which the scientific rationale for using genome editing technology may be recognized. In light of this, members of the Expert Panel on Bioethics reiterated the importance of ethical considerations in individual research and the review system for such considerations. The findings reported here by the Japanese Association of Medical Sciences served as one of the key factors leading to the Second Report that permits disease research using surplus embryos.

As described above, in the discussions of the Expert Panel on Bioethics regarding human genome editing, we were able to see the unprecedented multilateral and deep involvement of the research community, including new proposals from their standpoint and contributions to the discussion by organizing the research possibilities using genome editing from their experienced perspective.

#### Patient and Public

Patients and the public also played an important role in the discussions at the Expert Panel on Bioethics. The Task Force included members of the Japan Patient Association. Hearings were also held with the representatives of patient associations. Public dialogue events have also been actively held.

Hearings with patient association representatives were held twice in the third period when human embryo genome editing for disease research was the subject of discussion. This was groundbreaking since the previous hearings at the Expert Panel on Bioethics focused on sharing expertise with the so-called experts. First, at the 110th Expert Panel on Bioethics (July 27, 2018), a representative of the Japan Patients Association and a member of the Task Force made a presentation. The presenter pointed out the current situation in which biotechnology, including genome editing, has penetrated the general public without understanding its risks. At this time, a member of the Expert Panel pointed out that the government should seriously consider supporting funding and human resources in efforts to increase public understanding. Second, at the 16th Task Force (February 25, 2019), the president of the Japan Fabry Disease Patients and Family Association gave a presentation, in which he expressed expectations for research and treatment using genome editing from the perspective of patients and their families. He expressed his hopes for the careful and positive development of advanced medical technology, not only regarding genome editing, but also regarding medicine as a whole and even social equity and fairness. The following is a quote from the president’s remarks.^9^

“As is the case with our diseases, there are individual differences in symptoms even though they are the same disease. In the age of genomics, we will move away from conventional evidence-based medicine to so-called tailor-made, individualized medicine, and even the structure of medicine and methods of treatment will have to change. It can be said that the genome is changing not only the medical field but also the state of society. I hope that the way of medical care will be reexamined, one person at a time.”

“For the sake of patients and families who need genome editing technology, I hope that basic research using genome editing technology for hereditary and congenital diseases will be conducted carefully and without regret in the future, so that patients can enjoy fair and equitable medical services in the clinical research that follows. I would like to see such a state of affairs.”

In addition, several public dialogue events were held by the Cabinet Office (the administrative body supervising the Expert Panel on Bioethics), in cooperation with the National Museum of Emerging Science and Innovation (Miraikan), Science Council of Japan, and Japan Association for Bioethics. The Expert Panel on Bioethics members were closely involved in these events, and members who were involved in each event had a dialogue with the public and patients and reported their experiences to the Expert Panel on Bioethics. For example, at the event co-hosted with Miraikan, participants commented, “I felt the importance of discussing what kind of society we want to envision,” “It is a problem that many people do not know that this kind of discussion is taking place,” and “I hope that similar events will be held on the same theme on an ongoing basis.” This was reported at the 116th Expert Panel on Bioethics.^10^ In addition, one Expert Panel member participated in discussions at the Society of Intractable Disease Centers and the Japan Patients Association Board of Directors. Experiences from these occasions were also shared with the Expert Panel on Bioethics. The public and patients were involved and played a role in the discussions of the Expert Panel on Bioethics through these dialogue efforts.

## Discussion

In this study, we analyzed the discussion process of the ethics review system, a key tool of research governance, which was carried out by the Japanese Expert Panel on Bioethics. We found that stakeholder involvement in the ethics review system has been discussed intensively. It was also clear that various stakeholders, including patient representatives, were involved in the overall policy-making process of the Expert Panel on Bioethics.

### Where and How Should Ethics Review Boards be Established?

The discussion at the Expert Panel on Bioethics on the ethics review system for genome editing technologies began with a review of the various existing systems in Japan. As mentioned in the introduction of this paper, there are two types of Japanese ethics review systems in the existing guidelines: the IRB-only method and the two-step review method. In the two-step review, the national government, in addition to the research institution, is involved. Based on this, the key point is where and how ethics review boards should be established and who should be the stakeholders involved in the review activities.

In terms of the positioning of the review boards, there is a clear difference between IRBs and CRBs: IRBs are established for each research institution, while CRBs cover all research in the country and are separate from individual research institutions. In the discussion of the expert panel, it was pointed out that both of these two frameworks have advantages and disadvantages. Furthermore, by analogy with the two-step review, it was suggested that the IRB and the CRB be utilized in combination, and several proposals were discussed regarding the division of functions between the two. There were also discussions on the issue of who should establish and maintain the CRB. For example, should the national government establish one, or should academic societies take the lead?

Considering examples from other countries, ESCRO, in the USA, could be considered an IRB, and HFEA, in the UK, a CRB. These may be typical examples in terms of function. However, taking into account that a two-step review system is in place in Japan and that a combination of the two was discussed, there seems to be openness for the possible implementation of an atypical review system.

### Stakeholders in the Ethics Review System

How should stakeholders be involved in an ethics review system? First, our analysis confirmed that experts in the research community can play an important role in the ethics review system. It was proposed in the discussion that academic societies and the government should cooperate to establish an ethics review board in addition to the local review boards. This role of the research community was confirmed because of its interest in genome editing and the need for expertise in the ethical review of genome editing research.

Second, the pros and cons of the involvement of government agencies are highly debated. This is a basic argument for stakeholders in research governance. To govern genome editing research, some scholars focus on ‘soft’ forms of governance based on networks of multiple public and private stakeholders (Conley et al. 2020). Meanwhile, others point out the need for legitimate governance, including a combination of national and supranational legislative regulation or ‘hard’ law (Townsend 2020). Furthermore, it has been noted that genome editing requires global governance, the prerequisite for which is thorough regulation in each country (European Commission, Directorate-General for Research and Innovation 2021). Although the role of the government in research governance has been the focal point, China, for example, relies on local institutional ethics review boards for ethics review of research using gene editing technologies, and this system has been criticized (Peng et al. 2022). In the past, the importance of national oversight of human embryo research, which is similar to the United Kingdom’s HFEA and Canada’s Stem Cell Oversight Committee, was also pointed out in the USA (Kimmelman et al. 2006; Johnston 2005; Baylis and Robert 2006). In Japan, the issue of local review by institutional ethics review boards has also been highlighted. The question of how government agencies should be involved in resolving this issue was raised. The role of the government in the governance of emerging medical technologies, not just in the ethics review system, is potentially controversial in any country.

Public involvement in the ethics review system has not been explicitly addressed in the discussions analyzed here. However, in Japan, public participation in ethics reviews was taken for granted. The Guidelines for Research Using Gene-altering Technologies on Human Fertilized Embryos stipulate the requirement that the institution’s Ethical Review Committee shall have “a member who can provide opinions of the general public.” This phrase is widely found in other government guidelines that stipulate the requirements for institutional ethics review boards and is not specific to human embryo genome editing.

### Stakeholders in the Overall Policy-making Process

In this and the following sections, we discuss how stakeholders—the research community, government agencies, and the general public—were involved in the policy-making process of the Expert Panel on Bioethics.

First, in this discussion on genome editing, representatives from academic societies and patient groups participated in the Expert Panel on Bioethics and, in addition, the Expert Panel conducted a new public dialogue event (Fig. 1-B). In other words, it was demonstrated that the three stakeholders can convene to discuss the governance of emerging medical technologies in a forum established by government agencies. Discussions on research governance in a governmental framework are expected to directly lead to actual policies. The limitations of this framework are that the agenda and members of the Expert Panel on Bioethics were mainly selected by government agencies; however, thus far, no significant challenges have arisen. This time, some sharp points were raised, such as the pros and cons of having the national government involved in bioethics. In addition, discussions were held with representatives of academic societies and patient groups. These facts suggest that no significant bias existed even though the government officials managed the Expert Panel meetings.

Second, collaboration between the research community and government has been achieved. The collaboration was triggered by the research community’s active interest in the issue of genome editing through proposals and other means. To achieve sustained collaboration, government agencies were effective in providing a forum for discussion. The results of the collaboration are evident in the report of the Expert Panel on Bioethics (the Second Report and the Summary of Opinions), which is a government body. Some argue that the research community has a direct stake and should not govern itself in a democratic society (Jasanoff et al. 2019). A method of governance in which the research community and government agencies collaborate, as opposed to one in which the research community governs itself, can avoid these negative points, while taking advantage of the benefits of involvement.

Regarding the public, we found a certain degree of public involvement and the need to consider ways to engage diverse stakeholders comprising “the public” in the policy-making process, based on attributes that are relevant to the application of emerging medical technologies, rather than lumping them together as the general public.

### Interaction between the Research Community and Government

In Japan, there has been poor collaboration between the research community and the government. In the regulation of stem cell research, scientists, as key stakeholders, did not work well with policy-making bodies in the past (Kawakami et al. 2010). In genome editing, some argue that there is a division between the Japanese government and academic societies (Nakazawa et al. 2018). The involvement of research communities and government agencies in research governance varies from country to country. In some cases, legislatures have taken the lead in setting the framework for the regulation of human embryo research, as in the United Kingdom, France, and Germany, whereas in others, the research community has set the framework, as in the case of ESCRO in the USA.

Although there has been much discussion on genome editing in the research community worldwide, the status of regulation in each country is not uniform, as is the case with human embryo research. In China, to reduce unethical or illegal uses of emerging technologies, some argue that top-down regulation is crucial, i.e., that the governance of research should be under the jurisdiction of the State Council (Lei et al. 2019), and quick legal action has been taken by the legislature (Cao and Jia 2021). In the USA, because gene editing is a topic more remote from the long-standing abortion debate and has received less attention, its governance is likely to be undertaken by national and international scientific bodies, in collaboration with regulatory agencies, rather than by legislative or executive branches, as has recently been the case (Gabel and Moreno 2019).

In this Japanese case study, the research community and government agencies worked together on an ongoing basis, and their discussions were reflected in the report. The research community and government agencies have been noted to have strengths regarding governance (Gregorowius et al. 2017; Lei and Qiu 2020) and play complementary roles. Our results also showed that collaboration between the research community and government agencies has led to ideas for new review systems, suggesting that this can be a good approach to research governance.

In particular, we focused on the cases of four related academic societies and the Japanese Association of Medical Sciences. They belong to the biomedical research community. On the other hand, they are only one part of the professional community; there are other professional communities related to biomedicine. The humanities, social sciences, and industrial communities are also relevant to biomedicine. The Science Council of Japan includes members of the humanities and social sciences, who were involved in the development of the recommendations. However, none of the expert members who participated in the Expert Panel on Bioethics represented academic organizations in the humanities and social sciences. In addition, there was no notable involvement from the industry.

### How “the Public” Participated

#### The Public as Non-professional

The need for public participation has also been noted (National Academy of Sciences, Engineering, and Medicine 2017). In France, the Estates General of Bioethics was implemented, in conjunction with the revision of the bioethics law, which also addresses genome editing (États Généraux de la Bioéthique 2018). In the Netherlands, a public dialogue on heritable human genome editing (HHGE) has been attempted (van Baalen et al. 2021), and consequent changes in opinion have been studied (Houtman et al. 2022). In the United Kingdom, Austria, and Singapore, public consultations have been held on discussions regarding mitochondrial replacement technology (MRT) (Cohen et al. 2020), and public participation in advanced life science and technology is being attempted. In the UK, thorough public engagement in the HHGE discussion has been argued, as has been done for MRT (Adashi et al. 2020). In addition, among the criticisms of the research community leading the discussion, the need for public participation has been highlighted (Jasanoff et al. 2019; Blasimme 2019).

A new attempt at public participation in the Expert Panel on Bioethics was a public dialogue. Here, participants were widely invited from the public and asked for their opinions while introducing the scientific and social status of genome editing. In contrast to the research community’s deep involvement as experts, the public is expected to be non-experts. Although efforts have been previously made to reflect public perspectives in the discussions of the Expert Panel on Bioethics, they have not always been sufficient. One example is public comments, which have been conducted since the inception of the Expert Panel on Bioethics. However, public comments are limited to draft reports prepared by the Expert Panel on Bioethics, making it difficult to raise new issues from the standpoints of the public and patients. In addition, journalists have participated in the Expert Panel on Bioethics as expert members and expressed their opinions from a standpoint different from those of medical and legal expert members. In Japan, many members of the mass media have participated in government councils to reflect public opinion in government administration, and this relationship between government and journalism has been the subject of criticism owing to conflicts of interest (Amano 1993). The method of public dialogue is free from these restrictions and criticisms.

#### Roles of Each Sector with Some Expertise

“The public” actually includes a wide variety of positions, including the patient sector of diseases that may be affected by genome editing technologies and the religious sector, which has views on ethical issues. Each sector has its own expertise.

Patient sector involvement, apart from the patient and public involvement (PPI), deserves attention. Although PPI in research is useful, greater attention should be given to the question of who should be involved (Staley et al. 2021). In discussions on human embryo genome editing research, the involvement of patients with diseases that are likely to be affected by the application of genome editing should also be considered. The ways in which experts characterize the problems of disease and disability and define research agendas often reflect false assumptions about the affected people and ignore crucial dimensions of social context. Thus, greater diversity, which includes these patients, is needed in poising and framing key questions (Jasanoff et al. 2019). In the discussions of the Japanese Expert Panel on Bioethics, efforts were made to incorporate the views of patients, including participation of people from patient groups as members of the committee and holding of hearings from patients’ perspectives. Although these efforts were meaningful, no mechanism was created to incorporate their opinions on an ongoing basis.

Some argue that the research community should interact with religious scholars because religious beliefs shape our thinking on cloning, stem cells, and gene editing and affect our healthcare decisions and motivations for seeking treatment (Kalidasan and Das 2022). There are many examples of the involvement of the religious sector in national debates worldwide. At first, in the discussion on stem cells, the Japanese Expert Panel on Bioethics also had an Expert Panel member (who was not a religious person) specializing in religious studies. Experts also gave presentations on Buddhism (in the 13th meeting on March 15, 2002), Christianity (in the 13th meeting), and Oomoto, a relatively small sect of Shinto (in the 16th meeting on April 26, 2002). However, the main direction of the stem cell debate in Japan is not strongly influenced by religious groups (Kato 2005). This situation has not changed much during deliberations on human genome editing, which the present study focuses on. In Japanese discussions on genome editing, none of the panel members were experts in religious affairs. This fact probably reflects the state of social debate in Japan. This contrasts with what is seen in France (Mathieu 2020) and Italy (Corbellini 2007; Cattaneo and Corbellini 2011), where religion had a crucial impact on the policy-making process. The absence of a strong religious influence may have been one reason for the lack of extreme conflicts in the science and technology policy debate in Japan. However, there is potential for mutual understanding and collaboration. Scientists and religious leaders have been suggested to successfully work together to reach a mutual understanding and specific agreements on genetic technologies (Modell et al. 2019). The position of the religious sector in national committees needs further consideration, noting the trends in the social debate in each country.

Given the diversity of views among “the public,” it is necessary to consider how to engage stakeholders with distinctive views. Democratic approaches are likely to lead to fragmentation, rather than broad consensus building (Cavaliere et al. 2019). In Japan, although efforts have been made to actively include the views of the patient community, no attempt has been made to actively include the views of the religious sector, reflecting the state of social debate. Additionally, there is no set mechanism in place for the involvement of either position, and the situation remains under consideration. The patient and religious sectors discussed in this report are only two examples of the diverse members of “the public.” This issue needs to be further examined, not only in Japan, but also globally.

### Study Implications

Our work provides valuable insights into the roles of stakeholders in the governance of emerging medical technologies. First, we demonstrated that the involvement of the research community in research governance may not only take the form of self-regulation, but also collaboration with other stakeholders. This suggests that the role of the research community cannot be discussed without considering the relationships among stakeholders. This study also confirms that the general public is not uniform. Positions of significant interest vary depending on the technology being discussed. For effective discussion on the policy-making process, attention should be paid to the intensity of such interests. The degree to which stakeholders in the public are/should be involved will also vary depending on the social context and nature of the technology being discussed. For future work, the roles played by people in various positions, included in the so-called “the public,” need to be carefully examined, so that each stakeholder can play its maximum role in the governance of emerging technologies, such as genome editing.

### Study Limitations

The publicly available documents analyzed in this study do not contain records of individual out-of-meeting communications between the Secretariat of the Expert Panel on Bioethics and the Expert Panel members. Discussions in the research community and the general public are dealt with only to the extent that they are addressed by the Expert Panel on Bioethics. Therefore, we were unable to conduct an adequate analysis of the interactions among the various stakeholders in the research community, the outreach from the research community to the general public, and the spontaneous actions of the general public. To address these topics in future research, the relationships among the key stakeholders identified in this study (i.e., the government, research community, and general public) need to be examined.

## Conclusion

We have analyzed the deliberation process on the governance of human genome editing by the Expert Panel on Bioethics of Japan. Regarding the ethics review system for human embryo genome editing, the Panel discussed and debated what stakeholders should be involved in the ethics review system, the possibility of the research community being involved with its expertise, and the pros and cons of national oversight of individual research. Although discussions were directed at the Japanese ethics review system, we believe that our findings have general implications that would be useful for discussions in other countries. Furthermore, three stakeholders—the research community, government agencies, and public—could come together to discuss the governance of emerging medical technologies in a forum prepared by government agencies, namely the Expert Panel on Bioethics. This reaffirms that government agencies can play an active role in research governance discussions and enable various stakeholders to collaborate with each other and with government agencies. Another key finding is that various members of the public have engaged differently with the research community; they can play multiple roles, such as non-experts (lay public) or experts in their own particular field (patients as experts on their own diseases). The latter group can be involved in the policy-making process, as they have a wealth of insight specific to their respective circumstances. Therefore, the role of the public in the governance of emerging technologies needs to be examined carefully in future studies.

Our results may serve as a guide for countries and organizations aiming to establish governance on emerging medical technologies, even those beyond human genome editing technology, which includes the involvement of various stakeholders, including specialists, citizens, and patients. Analysis of the ethics review system may also serve as an example for those who need to establish an ethics review system.

## Data Availability

The datasets used during the current study are available from the corresponding author on reasonable request.

## Footnotes

1 The Expert Panel on Bioethics is one of the Expert Panels established in the Council for Science, Technology, and Innovation, Cabinet Office, Government of Japan, and is tasked with “developing guidelines for the handling of specific embryos based on Article 4, Paragraph 3 of the Law Concerning Regulation of Human Cloning Technology, etc., in order to respond to rapid developments in life science, and to conduct other investigations and studies related to bioethics.” Discussions are not limited to the “guidelines for the handling of specified embryos,” but cover a wide range of bioethical issues brought about by emerging medical technologies, etc. For example, extensive discussions have been held regarding reproductive medical research and regenerative medical research.

2 April 22, 2016 was also the date on which the Expert Panel on Bioethics released its “Interim Summary.”.

3 Statement from Four Societies on Human Genome Editing. https://www8.cao.go.jp/cstp/tyousakai/life/haihu98/shiryo2-1.pdf. Accessed 29 March 2023.

4 Minute of the 98th Expert Panel on Bioethics. https://www8.cao.go.jp/cstp/tyousakai/life/haihu99/shiryo1.pdf. Accessed 29 March 2023.

5 Classification of what the ethics review boards should be. https://www8.cao.go.jp/cstp/tyousakai/life/haihu101/siryo2.pdf. Accessed 29 March 2023.

6 The Second Report. https://www8.cao.go.jp/cstp/tyousakai/life/hitohaihoukoku2.pdf. Accessed 29 March 2023.

7 The Summary of Opinions. https://www8.cao.go.jp/cstp/tyousakai/life/haihu122/sanko1.pdf. Accessed 29 March 2023.

8 Minute of the 119th Expert Panel on Bioethics. https://www8.cao.go.jp/cstp/tyousakai/life/haihu120/siryo1.pdf. Accessed 29 March 2023.

9 Minute of the 16th Task Force. https://www8.cao.go.jp/cstp/tyousakai/life/tf/16kai/gijiroku.pdf. Accessed 29 March 2023.

10 “What do you think about research on human fertilized embryos?” Results report. https://www8.cao.go.jp/cstp/tyousakai/life/haihu116/sanko1.pdf. Accessed 29 March 2023.
